# Supplementary material for: Spine Metastasis Is Associated with the Development of Brain Metastasis in Non-Small-Cell Lung Cancer Patients
Source: Medicina (Kaunas). 2024 Jan 14;60(1):152. doi: 10.3390/medicina60010152 (PMC10820916; doi:10.3390/medicina60010152)
Supplement: Supplementary file 1 [file medicina-60-00152-s001.zip › medicina-2767685-supplementary.pdf]

Table S1 Association of bone metastasis with BM at diagnosis

	Univariate analysis		Multivariate analysis	
**Variables**	**OR (95% CI)**	**p value**	**OR (95% CI)**	**p value**
Age				
>69	reference	-		
≤69	1.28 (0.95–1.72)	0.109		
Gender				
Male	reference	-	reference	-
Female	1.46 (1.08–1.98)	0.015	0.72 (0.41–1.26)	0.250
Smoking history		-		
Ever	reference		reference	-
Never	1.77 (1.30–2.40)	<0.001	2.01 (1.13–3.60)	0.018
ECOG performance status				
0–1	reference	-	reference	-
≥2	1.34 (0.99–1.82)	0.057	1.29 (0.94–1.77)	0.118
Histology				
SQC	reference	-	reference	-
ADC	1.91 (1.27–2.87)	0.002	1.52 (0.97–2.40)	0.070
Others	1.15 (0.59–2.23)	0.679	1.05 (0.53–2.08)	0.883
EGFR mutation				
Negative	reference	-	reference	-
Positive	1.48 (1.08–2.02)	0.014	1.01 (0.70–1.47)	0.950
T category				
Tx–T1	reference	-	reference	-
T2	1.65 (0.78–3.48)	0.187	1.56 (0.72–3.39)	0.262
T3	1.87 (0.91–3.85)	0.089	1.68 (0.79–3.58)	0.181
T4	1.87 (0.95–3.67)	0.072	1.61 (0.79–3.29)	0.187
N category				
N0	reference	-	reference	-
N1	1.34 (0.74–2.42)	0.332	1.45 (0.78–2.70)	0.245
N2	1.51 (0.96–2.37)	0.073	1.71 (1.06–2.76)	0.028
N3	1.55 (1.06–2.29)	0.026	1.44 (0.94–2.18)	0.091
Bone metastasis				
No	reference	-	reference	-
Yes	2.31 (1.71–3.12)	<0.001	2.07 (1.50–2.84)	<0.001

ECOG PS, Eastern Cooperative Oncology Group; SQC, squamous cell carcinoma, ADC, adenocarcinoma; EGFR, Epidermal growth factor receptor

Table S2 Association of BM at diagnosis in patients with non-spine bone metastasis

	Univariate analysis		Multivariate analysis	
**Variables**	**OR (95% CI)**	**p value**	**OR (95% CI)**	**p value**
Age				
>69	reference	-		
≤69	1.28 (0.95–1.72)	0.109		
Gender				
Male	reference	-	reference	-
Female	1.46 (1.08–1.98)	0.015	0.74 (0.43–1.29)	0.284
Smoking history		-		
Ever	reference		reference	-
Never	1.77 (1.30–2.40)	<0.001	2.04 (1.15–3.62)	0.015
ECOG performance status				
0–1	reference	-	reference	-
≥2	1.34 (0.99–1.82)	0.057	1.37 (1.00–1.87)	0.050
Histology				
SQC	reference	-	reference	-
ADC	1.91 (1.27–2.87)	0.002	1.71 (1.09–2.68)	0.020
Others	1.15 (0.59–2.23)	0.679	1.15 (0.59–2.25)	0.686
EGFR mutation				
Negative	reference	-	reference	-
Positive	1.48 (1.08–2.02)	0.014	1.08 (0.75–1.56)	0.689
T category				
Tx–T1	reference	-	reference	-
T2	1.65 (0.78–3.48)	0.187	1.52 (0.71–3.28)	0.283
T3	1.87 (0.91–3.85)	0.089	1.61 (0.76–3.41)	0.210
T4	1.87 (0.95–3.67)	0.072	1.60 (0.79–3.23)	0.190
N category				
N0	reference	-	reference	
N1	1.34 (0.74–2.42)	0.332	1.73 (0.93–3.20)	0.083
N2	1.51 (0.96–2.37)	0.073	1.86 (1.16–2.98)	0.010
N3	1.55 (1.06–2.29)	0.026	1.61 (1.07–2.43)	0.024
Bone metastasis				
No	reference	-	reference	-
Yes	0.91 (0.58–1.43)	0.679	0.89 (0.55–1.42)	0.615

ECOG PS, Eastern Cooperative Oncology Group; SQC, squamous cell carcinoma, ADC, adenocarcinoma; EGFR, Epidermal growth factor receptor
